# Influence of Diabetes on Periapical Pathology in Treated and Untreated Teeth: A Cross-Sectional Comparison with Non-Diabetic Patients

**DOI:** 10.3390/jcm14113907

**Published:** 2025-06-02

**Authors:** Mihaela Sălceanu, Anca Melian, Cristian-Levente Giuroiu, Cristina Dascălu, Corina Concita, Claudiu Topoliceanu, Maria-Alexandra Mârţu

**Affiliations:** 1 Department of Odontology-Periodontology and Fixed Restorations, Faculty of Dental Medicine, University of Medicine and Pharmacy “Grigore T. Popa”, 700115 Iasi, Romania; mihaela.salceanu@umfiasi.ro (M.S.); anca.melian@umfiasi.ro (A.M.); corina-alexandra.concita@umfiasi.ro (C.C.); claudiu.topoliceanu@umfiasi.ro (C.T.); maria-alexandra.martu@umfiasi.ro (M.-A.M.); 2 Discipline of Medical Informatics and Biostatistics, Faculty of Dental Medicine, University of Medicine and Pharmacy “Grigore T. Popa”, 700115 Iasi, Romania; cristina.dascalu@umfiasi.ro

**Keywords:** apical periodontitis, endodontically treated teeth, diabetes type 2, HbA1c

## Abstract

The aim of this study was to explore diabetes mellitus type 2 as a risk factor in the prevalence of chronic apical periodontitis (CAP) in untreated and endodontically treated teeth. The second objective was to describe the correlation between the presence of periapical lesions and blood glucose/glycated hemoglobin levels among diabetic (DM) and non-diabetic patients with endodontically treated or untreated teeth with CAP. **Materials and methods:** This cross-sectional study was conducted on 90 patients (55 with DM, 35 without DM), admitted to the Oral and Maxillofacial Surgery Department of “St. Spiridon” Hospital in Iași, Romania. Endodontic and perapical status was evaluated using the Periapical Index (PAI) based on clinical and radiological evaluations with blood glucose levels and HbA1c analysis. Statistical analysis included data, correlations and regression analysis, and group comparisons using appropriate parametric or non-parametric tests. DM subjects had a higher mean value of untreated teeth with CAP (2.53 vs. 2.00) compared to the control group (*p* = 0.010) and a lower mean number of endodontically treated teeth without CAP (1.53 vs. 2.74) compared to the control group (*p* < 0.001). Diabetic patients also had a higher mean number of root-filled teeth with CAP (3.33 vs. 1.94; *p* < 0.001). **Conclusions:** There was a clear association between diabetes and oral pathology, with diabetic patients having fewer teeth, more untreated teeth, and a higher prevalence of periapical lesions. Elevated blood glucose and HbA1c levels indicated that poor metabolic control negatively impacts periapical healing and overall endodontic health.

## 1. Introduction

Chronic apical periodontitis (CAP) is a chronic inflammatory disease of the periapical tissues usually caused by persistent microbial invasion of the root canal system. Risk factors in chronic apical periodontitis include persistent microbial infection and microbial agents, inadequate root canal treatment, coronal leakage, poor oral hygiene practices, untreated dental caries, periodontal disease, dental traumas, missed canals, over-instrumentation or overfilling of the root canal, aging, and smoking status [1,2,3,4,5,6,7].

Although the direct cause of CAP is bacterial [8], the host’s systemic condition plays an important role in shaping the onset of disease as well as its progression and resolution [9]. After proper filling of root canals, the periapical tissue will resorb or heal depending entirely upon how well the host’s reparative response reacts to foreign matter [10]. So, the effect of endodontic treatment is to obliterate sources of bacterial antigens and toxins that form chronic inflamed tissue [11]. However, other systemic conditions render the host more susceptible to infection or impairs reparative response in periapical tissues. This context explains why so many endodontic cases fail [11]. A number of investigations have indicated a marked association between endodontic variables such as apical periodontitis or root-filled teeth (RFT) and non-devitalized RFT with systemic diseases of various kinds, including diabetes, cardiovascular diseases, extant tobacco habit, osteoporosis, or inherited coagulopathies. Specific diseases were also linked in some cases, which ranged from biological drugs to low birth weight (Table 1) [12]. Thus, the hyperinflammatory state of systemic disease with chronic inflammation leads to apical inflammation and may have an effect on its progression or prognosis [12]. Such associative findings cannot yet be regarded as a cause-and-effect relationship [13].

Among these systemic conditions, diabetes mellitus (DM) deserves special consideration because of its well-known effects on immune response, vascular function, and healing. Diabetes is a global public health issue, with a rising prevalence among all age groups [14,15]. It is associated with an increased risk of multiple oral pathologies such as periodontal disease, delayed post-operative healing, and endodontic problems. Impaired glycemic control has been associated with altered periapical responses, which can impact the development as well as the persistence of apical lesions. These effects are linked to reduced expression of mitogenic growth factors, increased levels of pro-inflammatory cytokines via epigenetic mechanisms, as well as the inhibition of cell expansion due to hyperglycemia and oxidative stress [16,17]. Oxidative stress influences significantly the pathogenesis of types 1 and 2 diabetes mellitus and their complications, due to main pathways of dysregulated glucose metabolism, oxidative injury to pancreatic β-cells, and endothelial dysfunction [18,19,20].

Interest in the oral-systemic health connection in diabetes has grown, yet only a few studies have investigated the association between glycemic status and the prevalence of apical periodontitis in endodontically treated and untreated teeth. Moreover, there are very few comparative studies regarding diabetic vs. non-diabetic populations to show differences in periapical health. A variety of oral conditions are associated with diabetes mellitus, the most frequently investigated being periodontal disease, followed by dental caries and tooth loss [21,22,23,24,25]. Although less extensively studied, the relationship between diabetes mellitus and periapical pathology is significant and warrants further attention from research groups. Literature data indicate that endodontic therapy improved the healing of CAP, glycemic control, and systemic inflammation in patients with DM and/or CAP. However, the researchers did not find a continued decreasing of HbA1c in the short term [26,27]. Diabetes effects on the decrease of the periapical healing rate are explained by immunological and microvascular alterations characteristic of diabetes, which support sustained local inflammation and hinder periapical tissue regeneration. Therefore, diabetes mellitus—particularly in its uncontrolled metabolic forms—represents a systemic condition with major negative impact on the progression and prognosis of chronic periapical lesions. Hyperglycaemia stimulates bone resorption, inhibiting osteoblastic differentiation and reducing bone regeneration processes [28]. However, an accurate mechanism through which hyperglycemia contributes to periodontal destruction remains incompletely understood. Advanced glycation end products (AGEs) as well as altered collagen metabolism, or impaired immune function (affecting polymorphonuclear leukocyte activity) may facilitate bacterial presence in the periapical tissues [29,30]. The accumulation of AGEs contributes to prolonged hyperglycemia and chronic inflammatory states, characterized by elevated secretion of proinflammatory cytokines, such as tumor necrosis factor-alpha and prostaglandin E2 [31]. Increased collagenase activity, coupled with reduced collagen synthesis, adversely affects collagen turnover, compromising both lesion healing and the integrity of periodontal tissues [32]. Scientific evidence has demonstrated that diabetic patients are at increased risk of an impaired immune response to periapical pathogens, as well as heightened susceptibility to residual lesions following endodontic treatment [33,34].

We aimed to explore diabetes mellitus type 2 as a risk factor of the prevalence of periapical lesions in untreated and root-filled teeth. The second objective of the study was to describe the correlation between the presence of periapical lesions and blood glucose/glycated hemoglobin levels, comparing glycemia and HbA1c values among patients with endodontically treated or untreated teeth with CAP.

## 2. Materials and Methods

The study sample included 90 patients (55 with diabetes mellitus [DM] and 35 without DM); socio-demographic parameters: 49 men—31 patients with DM and 18 without DM; 41 women—24 subjects with DM and 17 without DM; mean age was 60.13 years (range: 39–88 years). The patients were randomly selected from among those originating from the Northeastern region of Moldova (Romania), hospitalized at “St. Spiridon” Hospital in Iași, in the Department of Oral and Maxillofacial Surgery. This study respected the principles of Declaration of Helsinki and received approval from the Ethics Department of the University of Medicine and Pharmacy “Grigore T. Popa” Iasi (Romania) (No. 334/16 July 2023). Informed consent was provided by all patients participating in the study by signing a consent form approved by the ethics committee of “Grigore T. Popa” University of Medicine and Pharmacy in Iași.

The subjects were diagnosed according to the criteria issued by the American Diabetes Association. The subjects included in the study group confirmed the use of the pharmacological treatment prescribed by their diabetologist for DM. Upon admission to the Oral and Maxillofacial Surgery department, in addition to routine general and local investigations, both fasting blood glucose and glycated hemoglobin (HbA1c) were assessed.

Digital panoramic radiographs of the patients’ teeth were taken in order to identify the endodontic and periapical status. Two radiological technicians used a digital orthopantomograph (CRANEX D CEPH, DC-type generator, operating frequency 40 kHz, SOREDEX, Tuusula, Finland) to obtain panoramic radiographs. Furthermore, all teeth of the patients included in this study were recorded clinically and statistically, except for the third molars. The following information was recorded to create a structured profile for each subject: (i) The number and location of untreated teeth with diagnosed CAP; (ii) The number and location of endodontically treated teeth without diagnosed CAP; (iii) The number and location of endodontically treated teeth with diagnosed CAP. The periapical status of the recorded teeth was assessed using the Periapical Index (PAI) described by Dag Ørstavik in 1986. Two dental radiology specialists identified and recorded the teeth included in the study groups using the Periapical Index (PAI) scoring system. Initially, the examiners underwent a calibration process. This simulation involved the evaluation of 50 radiographs, including healthy teeth, teeth with chronic apical periodontitis (CAP) ranging from PAI scores of 2 to 5, as well as root-filled teeth with or without CAP. Reproducibility was assessed by assigning PAI scores to all patients at an interval of 3 months after baseline. Before the second session of radiographic scoring, both observers were recalibrated by reviewing 100 radiographic images. All PAI scores over 1 were considered indicative of periapical pathology. All collected data were entered into Excel (Microsoft Corporation, Redmond, WA, USA).

To investigate the relationship between the number of present teeth, the number of untreated teeth without periodontitis, and blood glucose levels, including glycated hemoglobin (HbA1c), a correlation study and linear regression analysis was conducted. The interpretation of the correlation coefficients was based as follows:

- a correlation coefficient between 0.00 and 0.25 indicates no or very weak correlation;
- a coefficient between 0.25 and 0.50 indicates a fair correlation;
- a coefficient between 0.50 and 0.75 indicates a moderate to good correlation;
- a coefficient above 0.75 indicates a very strong correlation.

The correlation between the presence of periapical lesions and blood glucose/glycated hemoglobin levels across different groups was assessed by comparing glycemia and HbA1c values among patients with varying degrees of periapical lesion involvement, whether treated or not. The classification by degree of involvement was based on the following categories: 0–one tooth; two–three teeth; four or more teeth. Since this classification resulted in three study groups, and because neither the blood glucose values nor the HbA1c values followed a normal distribution, the non-parametric Kruskal–Wallis test was used for comparison between the groups. Statistical analysis was performed using SPSS version 20.0. The first step included descriptive statistics, where frequency distributions were calculated for the qualitative variables, and descriptive parameters were computed for the quantitative variables. Numerical variables were tested using the Kolmogorov–Smirnov test to assess their conformity with the normal distribution, which determined the appropriate statistical tests to be used in the analytical phase. In the analytical phase, comparisons between study groups were made using the Student’s *t*-test and ANOVA, when the assumption of normal distribution was met. In cases where normality was not confirmed, non-parametric tests such as the Mann–Whitney U test and Kruskal–Wallis test were applied. Frequency distributions of categorical variables were compared among groups using the Chi-square test. To explore relationships between numerical variables, Pearson correlation coefficients were calculated, and corresponding linear regression models were developed.

## 3. Results

In the test group (non-DM patients), the mean blood glucose level was 90.72 ± 9.825, while the mean glycated hemoglobin value was 4.56 ± 0.634. The mean glycated hemoglobin value (HbA1c) in the DM patients (8.0038 ± 1.69421) as well as mean blood glucose level (201.44 ± 81.227) were significantly higher when compared to non-diabetic patients (*p* < 0.001 **) (Table 1).

The patient group was investigated regarding the presence of periapical lesions, comparing diabetic patients with healthy subjects. The descriptive statistical results highlighting statistically significant differences between diabetic patients and healthy subjects, indicating a notable deterioration in oral health status, are exposed in Table 1. The difference between the test group and the control group in terms of teeth status and the presence of CAP are statistically significant (*p* < 0.05). The mean number of untreated teeth having CAP was found to be significantly higher in test group (2.53 ± 1.470) as compared to the control group (2.00 ± 0.939) with a Chi-square value of 16.747 and a *p* value = 0.010, which was statistically significant. The test group had statistically and significantly lower mean number of endodontically treated without apical periodontitis (CAP) (1.53 ± 1.419), as compared to the control group (2.74 ± 1.836), with a Chi-square value of 32.804 and a *p*-value < 0.001. Likewise, the number of endodontically treated teeth with CAP was significantly increased in the test group (3.33 ± 2.028) than in the control group (1.94 ± 1.123), *p* value < 0.001 (Table 2).

In diabetic patients, a statistically significant, directly proportional, fair correlation was observed between blood glucose levels and the number of untreated teeth without CAP. In the control group, a null and statistically non-significant correlation was found between blood glucose levels and both the number of teeth present and the number of untreated teeth without CAP (Table 3).

In diabetic patients, an acceptable, directly proportional, and statistically significant correlation was observed between glycated hemoglobin (HbA1c) levels and the number of untreated teeth without chronic apical periodontitis. In contrast, the control group showed a null and statistically non-significant correlation between HbA1c levels and this variable (Table 4).

Among diabetic female patients, a good directly proportional, and statistically significant correlation was found between blood glucose levels and the number of untreated teeth without CAP. In contrast, among male patients, no statistically significant correlation was found between blood glucose levels and the number of untreated teeth without CAP (Table 5).

Similarly, in diabetic female patients, a good, directly proportional, and statistically significant correlation was identified between HbA1c levels and the number of untreated teeth without CAP. On the other hand, in diabetic male patients, glycated hemoglobin showed a very weak correlation with the number of untreated teeth without CAP, directly proportional, and statistically non-significant (Table 6).

In diabetic patients living in rural areas, blood glucose levels showed an acceptable but statistically non-significant correlation with the number of untreated teeth without CAP. In urban diabetic patients, blood glucose levels weakly but still significantly correlated with the number of untreated teeth without CAP (Table 7).

In rural diabetic patients, a moderate, directly proportional, and statistically significant correlation was found between HbA1c levels and the number of untreated teeth without CAP. On the other hand, in urban diabetic patients, glycated hemoglobin correlation with the number of untreated teeth without chronic apical periodontitis was very weak, directly proportional, and statistically non-significant (Table 8).

In contrast, in the rural subgroup of the control group, a null correlation was identified between HbA1c levels and the number of untreated teeth without CAP. Among urban control patients, no statistically significant correlations were found between glycated hemoglobin levels and the number of untreated teeth without CAP (Table 9).

## 4. Discussion

Our study aimed to evaluate the correlation between glycemic control assessed through glycemia levels, glycated hemoglobin (HbA1c) levels, and CAP prevalence. Also, we aimed to enhance understanding of systemic metabolic status effects on periapical disease patterns and endodontic outcomes by comparing diabetics (DM) and non-diabetics, and treated and untreated teeth, respectively. The practical relevance of this study is linked by literature data reporting variable CAP prevalence in different populations, need for root canal treatment (assessed to 30–50% of the European population), and the prevalence of CAP in endodontically treated teeth (between 30 and 65% of the endodontically treated teeth were diagnosed with CAP) [35]. The mean number of untreated teeth without periapical lesions was significant lower in the diabetic group compared to the control group. This outcome may also be explained by the lower number of remaining teeth in the oral cavity among diabetic patients compared to controls. Conversely, the mean number of untreated teeth with detectable periapical lesions was significantly higher in the diabetic group compared to the control group. Regarding root-filled teeth without CAP, the mean value was significantly lower in the diabetic group than in the controls. Additionally, the mean number of root-filled teeth with CAP was higher in the diabetic group compared to the control group. This was also correlated with a significantly higher number of untreated teeth with CAP as well as a higher number of root-filled teeth with CAP. It is also noteworthy that the number of root-filled teeth without CAP was significantly lower in diabetic patients compared to healthy subjects. Regarding blood glucose levels in relation to the number of untreated teeth with CAP in diabetic patients: No statistically significant differences were found, neither in the overall sample, nor when comparing by area of residence or among male patients. In the overall group, a slight decrease in average blood glucose values was observed as the number of untreated teeth with CAP increased—this trend was also noted among diabetic patients living in urban areas. Although the differences were not statistically significant, among male patients, the highest average blood glucose level was recorded in those with two or three untreated teeth diagnosed with CAP, while in rural areas, patients with over four untreated teeth with CAP had the highest glycemia mean values. Our results support similar research focused on bilateral relationship between diabetes and periapical pathology. A research group reviewed available evidence on the association between diabetes and the presence of CAP in root-filled teeth. Uni- and multivariate analysis of diabetic and non-diabetic patients calculated an OR 1.42 for higher risk of CAP in endodontically treated teeth [35]. A study that was conducted by a research group has demonstrated that 12 months after the tooth was undertaken endodontically treated patients can, in their view, expect a significant reduction in PAI score. However, we found significantly less periapical healing in the group of diabetics (43%) than in the non-diabetic control group for the same follow-up period [36]. A meta-analysis found OR 2.44 with a significant higher risk of extracted endodontically treated teeth in diabetics when compared with nondiabetic patients. A research group reported a significant increase of the frequency of extracted endodontically treated teeth in diabetic patients [37]. A study of diabetes impact on the prevalence of endodontic therapy found that diabetic patients had 6.1% of teeth with root canal treatment, while in healthy patients this percentage dropped to 3% (OR = 1.7). A regression analysis confirmed that patients with poor glycemic control were more likely to experience persistent apical periodontitis after treatment (OR = 4.78). Diabetes mellitus delays periapical healing, while periapical lesions may contribute to systemic inflammation in diabetic patients [38]. Our results supported by literature show that dentists must take into account the high prevalence of root-filled teeth with chronic periapical lesions in diabetic patients, leading to the conclusion that diabetes mellitus as an important preoperative prognostic factor in root canal treatment [35,36,37,38]. In our study, in diabetic patients, both blood glucose and HbA1c levels showed statistically significant, directly proportional correlations with the number of untreated teeth without chronic apical periodontitis (CAP), particularly among women and individuals living in rural areas. By contrast, these associations were either absent or statistically non-significant in the non-diabetic (control) group, regardless of gender or residence. Notably, diabetic male patients and urban residents exhibited weaker and often non-significant correlations, suggesting that gender and environmental factors may influence the relationship between glycemic status and periapical health. In control subjects, correlations between glycemic markers and the number of untreated teeth without CAP were predominantly weak, inverse, and statistically non-significant, further highlighting the distinct impact of diabetes on oral inflammatory conditions. Higher HbA1c levels and poor glycemic control in type 2 diabetic patients are correlated with poor periapical status and higher prevalence of apical periodontitis in endodontically treated teeth [29,35].

Higher prevalence of CAP patients with DM were reported when compared to non-diabetic subjects [39,40,41,42,43]. These findings are supported by clinical studies showing that periapical radiolucencies were observed in only 48% of patients with high plasma glucose concentrations (90–110 mg/dL), compared to the higher prevalence of patients (74%) with lower glycemic values (70–89 mg/dL) [44]. Other studies have identified an increase in periapical radiolucencies following endodontic treatment in patients with poorly controlled [45,46]. Glucose metabolism may accelerate osteoclasts differentiation, and bone in diabetic patients exhibits low numbers of osteoblasts [47]. The growth and differentiation of osteoblasts are particularly sensitive to osmotic stress, which may explain the bone mass loss associated with diabetes mellitus [48]. Decompensated diabetic patients often experience hypercalciuria and bone loss due to the decrease of bone formation processes associated to the increase of bone resorption [49]. Our findings contradict some published data, such as the cross-sectional study by Marotta et al. (2012) [50], where the authors aimed to assess the prevalence of periapical periodontitis and frequencies of endodontic treatment in type 2 diabetic versus non-diabetic Brazilian adult patients. They found that apical lesions occurred most frequently in teeth which had not been treated, and the prevalence among diabetics was significantly higher when compared to controls. When examining for untreated teeth alone, rates before they began receiving insulin shots were 10% for those in the DM groups versus 7% among non-DM subjects. No significant differences were found as follows: in the frequency of apical periodontitis on root-filled teeth; the number of teeth per jaw ridge arch; the number of teeth treated per person; and/or percentage with one or more apical lesions that have formed in chronic alveolar bone inflammation resulting from trauma at these sites [50]. When López-López et al. (2011) compared the prevalence of root canal–treated teeth with CAP in diabetics vs. control populations, they stratified their samples by age and sex and divided the diabetic subgroup into groups based on HbA1c levels (≤6.5%). Their data showed that the ratio of root-filled teeth with CAP was twice as high in diabetics versus non-diabetics without statistically significant differences [51].

One limitation of this study is the potential selection bias resulting from the hospital-based sample. As the participants were recruited from a single oral and maxillofacial surgery department, they may not fully represent the general population. Patients seeking hospital-based dental care might have more advanced oral or systemic conditions, potentially influencing the prevalence and severity of periapical pathology observed in our diabetic and non-diabetic groups. Another limitation is related to the simple fact that confounding factors such as whether one is a smoker or their level of oral hygiene were not taken into consideration. As we pointed out earlier, these variables are related to glycemic control and oral health outcomes, so their absence might have led to residual confounding. Such residual confounding can be avoided only if future studies have larger sample sizes and perform multivariate adjustment. Although the two radiology examiners underwent calibration sessions and repeated assessments, inter-rater reliability using kappa statistics was not formally reported. This omission limits the ability to fully assess consistency in PAI scoring, although the calibration process was designed to enhance reproducibility. A further limitation lies in the overlap of HbA1c values between diabetic and non-diabetic patients. The study did not apply predefined categories of glycemic control (e.g., controlled vs. uncontrolled diabetes), which may have obscured differences in periapical outcomes based on metabolic status. This study included several subgroup analyses (e.g., by sex and place of residence), which were exploratory in nature and not adjusted for multiple comparisons. Therefore, the findings from these analyses should be interpreted cautiously due to the potential for errors. In future studies, adjustment methods such as Bonferroni correction should be applied to limit spurious associations when conducting multiple hypothesis testing.

Considering the third place of DM in the range of chronic medical conditions among patients from dental cabinets, endodontic specialists must be aware by the increased risk of CAP and their negative impact on glycemic control [35]. Relevant therapeutic strategies must include patient education, improved glycemic and oral health management, inflammation control, and avoidance of tobacco and alcohol use [52,53,54]. Consequently, interdisciplinary healthcare teams should inform diabetic patients about their elevated risk for oral diseases and encourage regular dental checkups, integrating systemic and oral health approaches for comprehensive patient care [55,56,57,58,59,60]. Considering that diabetic patients are often unaware of their increased risk for oral complications, healthcare professionals from both general medicine and dentistry must develop public education programs to raise awareness of the oral manifestations of diabetes and their implications for oral health [61,62,63,64,65,66,67]. Due to present limitations of the study, the observed results should be taken with consideration.

## 5. Conclusions

There is a relationship between diabetes and poor oral health. Diabetic patients have fewer teeth, more untreated teeth, and a higher prevalence of periapical lesions. The total number of endodontically treated teeth with CAP was greater in diabetics.

The outcome of endodontic treatment appears less favorable in diabetics. Furthermore, they had significantly fewer endodontically treated teeth without CAP, which may indicate a reduced healing capacity or greater susceptibility to the recurrence of chronic apical lesions.

Higher blood glucose and HbA1c levels are associated with more severe endodontic disease, suggesting that poor metabolic control is detrimental to periapical healing and overall endodontic health, providing further evidence supporting systemic-local interaction between hyperglycemia and periapical inflammation.

## Figures and Tables

**Table 1:** Mean blood glucose and HbA1c levels.

	Non-DM Patients N = 35	DM Patients N = 55	*p*-Value
	m ± SD	m ± SD	
Glucose	90.72 ± 9.825	201.44 ± 81.227	<0.001 *
HbA1c	4.560 ± 0.634	8.004 ± 1.694	<0.001 *

* Statistically significant.

**Table 2:** The results of the Chi-square test, the significance threshold, the mean value, and the standard deviation in the study and control groups.

Test Group vs. Control Group	*p*	Mean Value ± Standard Deviation	
		Test Group (DM)	Control Group (Non-DM)
Untreated teeth with CAP	*p* = 0.010 *	2.53 ± 1.470	2.00 ± 0.939
Endodontically treated teeth without CAP	*p* = 0.000 *	1.53 ± 1.419	2.74 ± 1.836
Endodontically treated teeth (CAP)	*p* = 0.000 *	3.33 ± 2.028	1.94 ± 1.123

* Statistically significant.

**Table 3:** Correlation coefficients and regression lines between blood glucose levels and the number of teeth present in the oral cavity, as well as the number of untreated teeth without chronic apical periodontitis, in diabetic patients and the control group.

Group	Correlated Parameters	Pearson Correlation Coefficient			Equation of the Regression Line Y = A ∗ X + B	ANOVA Test for Model Fitting
		R	R^2^	Std. Error		*p*
DM	Number of teeth present in the oral cavity vs. Blood glucose	0.370	0.137	75.797	Y = 3.913 ∗ X + 139.107	0.000
	Number of untreated teeth without apical periodontitis vs. Blood glucose	0.254	0.064	78.928	Y = 2.964 ∗ X + 176.003	0.007
CONTROL	Number of teeth present in the oral cavity vs. Blood glucose	0.000	0.000	9.898	Y = 0.001 ∗ X + 90.712	0.997
	Number of untreated teeth without apical periodontitis vs. Blood glucose	0.074	0.005	9.871	Y = −0.108 ∗ X + 91.834	0.545

**Table 4:** Correlation coefficients and regression line between glycated hemoglobin values and the number of teeth present in the oral cavity, as well as the number of untreated teeth without chronic apical periodontitis in diabetic patients/control group.

Group	Correlated Parameters	Pearson Correlation Coefficient			Equation of the Regression Line Y = A ∗ X + B	ANOVA Test for Model Fitting
		R	R^2^	Std. Error		*p*
DM	Number of teeth present vs. Glycated hemoglobin	0.392	0.153	1.56598	Y = 0.086 ∗ X + 6.629	0.000
	Number of untreated teeth without apical periodontitis vs. Glycated hemoglobin	0.258	0.067	1.64432	Y = 0.063 ∗ X + 7.464	0.006
CONTROL	Number of teeth present vs. Glycated hemoglobin	0.034	0.001	0.63802	Y = −0.003 ∗ X + 4.615	0.780
	Number of untreated teeth without apical periodontitis vs. Glycated hemoglobin	0.079	0.006	0.63642	Y = −0.007 ∗ X + 4.633	0.521

**Table 5:** Correlation coefficients and regression line between blood glucose values and the number of teeth present in the oral cavity, as well as the number of untreated teeth without chronic apical periodontitis, in male and female diabetic patients.

DM Group	Correlated Parameters	Pearson Correlation Coefficient			Equation of the Regression Line Y = A ∗ X + B	ANOVA Test for Model Fitting
		R	R^2^	Std. Error		*p*
Gender FEMALE	Number of teeth present in the oral cavity vs. Blood glucose	0.492	0.242	81.769	Y = 6.680 ∗ X + 117.703	0.000
	Number of untreated teeth without apical periodontitis vs. Blood glucose	0.434	0.188	84.604	Y = 6.615 ∗ X + 168.909	0.002
Gender MALE	Number of teeth present in the OC vs. Blood glucose	0.308	0.095	63.564	Y = 2.445 ∗ X + 144.704	0.015
	Number of untreated teeth without apical periodontitis vs. Blood glucose	0.143	0.021	66.117	Y = 1.250 ∗ X + 172.633	0.266

**Table 6:** Correlation coefficients and regression line between glycated hemoglobin values and the number of teeth present in the oral cavity, as well as the number of untreated teeth without chronic apical periodontitis, in male and female diabetic patients.

DM Group	Correlated Parameters	Pearson Correlation Coefficient			Equation of the Regression Line Y = A ∗ X + B	ANOVA Test for Model Fitting
		R	R^2^	Std. Error		*p*
Gender FEMALE	Number of teeth present in the oral cavity vs. Glycated hemoglobin	0.502	0.252	1.47695	Y = 0.124 ∗ X + 6.602	0.000
	Number of untreated teeth without apical periodontitis vs. Glycated hemoglobin	0.449	0.202	1.52619	Y = 0.124 ∗ X + 7.539	0.001
Gender MALE	Number of teeth present in the oral cavity vs. Glycated hemoglobin	0.351	0.123	1.47925	Y = 0.066 ∗ X + 6.506	0.005
	Number of untreated teeth without apical periodontitis vs. Glycated hemoglobin	0.169	0.028	1.55708	Y = 0.035 ∗ X + 7.250	0.190

**Table 7:** Correlation coefficients and regression line between blood glucose values and the number of teeth present in the oral cavity, as well as the number of untreated teeth without chronic apical periodontitis, by place of residence, in diabetic patients.

DM Group	Correlated Parameters	Pearson Correlation Coefficient			Equation of the Regression Line Y = A ∗ X + B	ANOVA Test for Model Fitting
		R	R^2^	Std. Error		*p*
Residence RURAL	Number of teeth present in the oral cavity vs. Blood glucose	0.471	0.221	82.579	Y = 5.787 ∗ X + 85.273	0.011
	Number of untreated teeth without apical periodontitis vs. Blood glucose	0.336	0.113	88.147	Y = 4.357 ∗ X + 145.628	0.080
Residence URBAN	Number of teeth present in the oral cavity vs. Blood glucose	0.363	0.132	72.991	Y = 3.789 ∗ X + 146.939	0.001
	Number of untreated teeth without apical periodontitis vs. Blood glucose	0.247	0.061	75.905	Y = 2.911 ∗ X + 181.124	0.026

**Table 8:** Correlation coefficients and regression line between glycated hemoglobin values and the number of teeth present in the oral cavity, as well as the number of untreated teeth without chronic apical periodontitis, by place of residence, in diabetic patients.

DM Group	Correlated Parameters	Pearson Correlation Coefficient			Equation of the Regression Line Y = A ∗ X + B	ANOVA Test for Model Fitting
		R	R^2^	Std. Error		*p*
Residence RURAL	Number of teeth present in the oral cavity vs. Glycated hemoglobin	0.490	0.240	1.94152	Y = 0.143 ∗ X + 5.482	0.008
	Number of untreated teeth without apical periodontitis vs. Glycated hemoglobin	0.386	0.149	2.05546	Y = 0.119 ∗ X + 6.850	0.043
Residence URBAN	Number of teeth present in the oral cavity vs. Glycated hemoglobin	0.340	0.116	1.41598	Y = 0.068 ∗ X + 6.908	0.002
	Number of untreated teeth without apical periodontitis vs. Glycated hemoglobin	0.174	0.030	1.48274	Y = 0.040 ∗ X + 7.621	0.118

**Table 9:** Correlation coefficients and regression line between glycated hemoglobin values and the number of teeth present in the oral cavity, as well as the number of untreated teeth without chronic apical periodontitis, by place of residence, in patients from the control group.

DM Group	Correlated Parameters	Pearson Correlation Coefficient			Equation of the Regression Line Y = A ∗ X + B	ANOVA Test for Model Fitting
		R	R^2^	Std. Error		*p*
Residence RURAL	Number of teeth present in the oral cavity vs. Glycated hemoglobin	0.182	0.033	0.58011	Y = 0.017 ∗ X + 4.024	0.394
	Number of untreated teeth without apical periodontitis vs. Glycated hemoglobin	0.066	0.004	0.58871	Y = 0.005 ∗ X + 4.287	0.759
Residence URBAN	Number of teeth present in the oral cavity vs. Glycated hemoglobin	0.041	0.002	0.64869	Y = −0.004 ∗ X + 4.733	0.787
	Number of untreated teeth without apical periodontitis vs. Glycated hemoglobin	0.061	0.004	0.64803	Y = −0.006 ∗ X + 4.723	0.689

## Data Availability

Data supporting reported results can be provided by the corresponding authors upon reasonable request.
